# 

**Published:** 1920-10

**Authors:** 


### ANNOUNCEMENTS

*Annual Meeting Ohio State Dental Society.* This meeting will be held November 30th to and including December 2d, in Memorial Hall, Columbus. An unusually attractive program is being prepared.—F. R. Chapman, Secretary.

---

*U. S. Civil Service Examinations* will be closed for dentists November 23, 1920. Salary, $1,500 with extras. Secure application blanks and information at once.

---

*Alumni University of Iowa* will hold its dental clinics November 12, 1920 in College Building, Iowa. City.

